# Epiploic Appendagitis Masquerading as Acute Appendicitis: A Report of Two Cases

**DOI:** 10.7759/cureus.10689

**Published:** 2020-09-28

**Authors:** Aaliya F Uddin, Gautam Menon, Arathi Menon, Abdalla Saad Abdalla Al-Zawi, Jay Menon

**Affiliations:** 1 General Surgery, Basildon and Thurrock University Hospital, Basildon, GBR; 2 Acute Medicine, Mid and South Essex NHS Foundation Trust, Chelmsford, GBR; 3 Radiology, Royal Free Hospital, London, GBR; 4 Breast Surgery, Anglia Ruskin University, Chelmsford, GBR; 5 Breast Surgery, Basildon and Thurrock University Hospital, Basildon, GBR; 6 General Surgery, Mid and South Essex NHS Foundation Trust, Basildon, GBR; 7 Vascular Surgery, Basildon and Thurrock University Hospital, Basildon, GBR

**Keywords:** appendicitis, epiploic appendagitis, diagnostic laparoscopy, acute abdominal pain

## Abstract

Epiploic appendagitis (EA) is a rare clinical entity caused by an inflammatory/ischemic process involving the serosal outpouchings of the colon. Its clinical presentation of acute, localised, lower abdominal pain often mimics more common conditions like diverticulitis or appendicitis. The diagnosis of EA is challenging due to the lack of pathognomic clinical features. The definitive diagnosis primarily relies on cross-sectional imaging modalities like abdominal ultrasound or computed tomography (CT). Being a benign and self-limiting condition, it can be managed conservatively with analgesic and anti-inflammatory drugs. We present two cases to highlight EA as an important differential diagnosis for cases of acute lower abdominal pain, crucial to prevent unnecessary antibiotic therapy and surgical interventions.

## Introduction

Epiploic appendagitis (EA) is a benign, mostly self-limiting, inflammatory/ischemic disorder of the epiploic appendages, which are fat-filled serosal outpouchings of the colon. Clinically, it often mimics the more common causes of acute lower abdominal pain such as acute appendicitis or acute diverticulitis [1-3]. Owing to its rarity and non-specific clinical presentation, the clinical diagnosis of EA is challenging. There has been a growing number of case reports of EA in the surgical literature following the increasing usage of abdominal computed tomography (CT) in the investigation of acute abdominal pain and the increase in diagnostic laparoscopic procedures. Additionally, the management is mostly conservative, with diagnostic laparoscopy undertaken in cases with unsure diagnosis or complications. We present here two unique cases to highlight this condition as an important differential diagnosis of acute lower abdominal pain, with an objective to avoid unnecessary surgical interventions when possible.

## Case presentation

**Figure 1:**
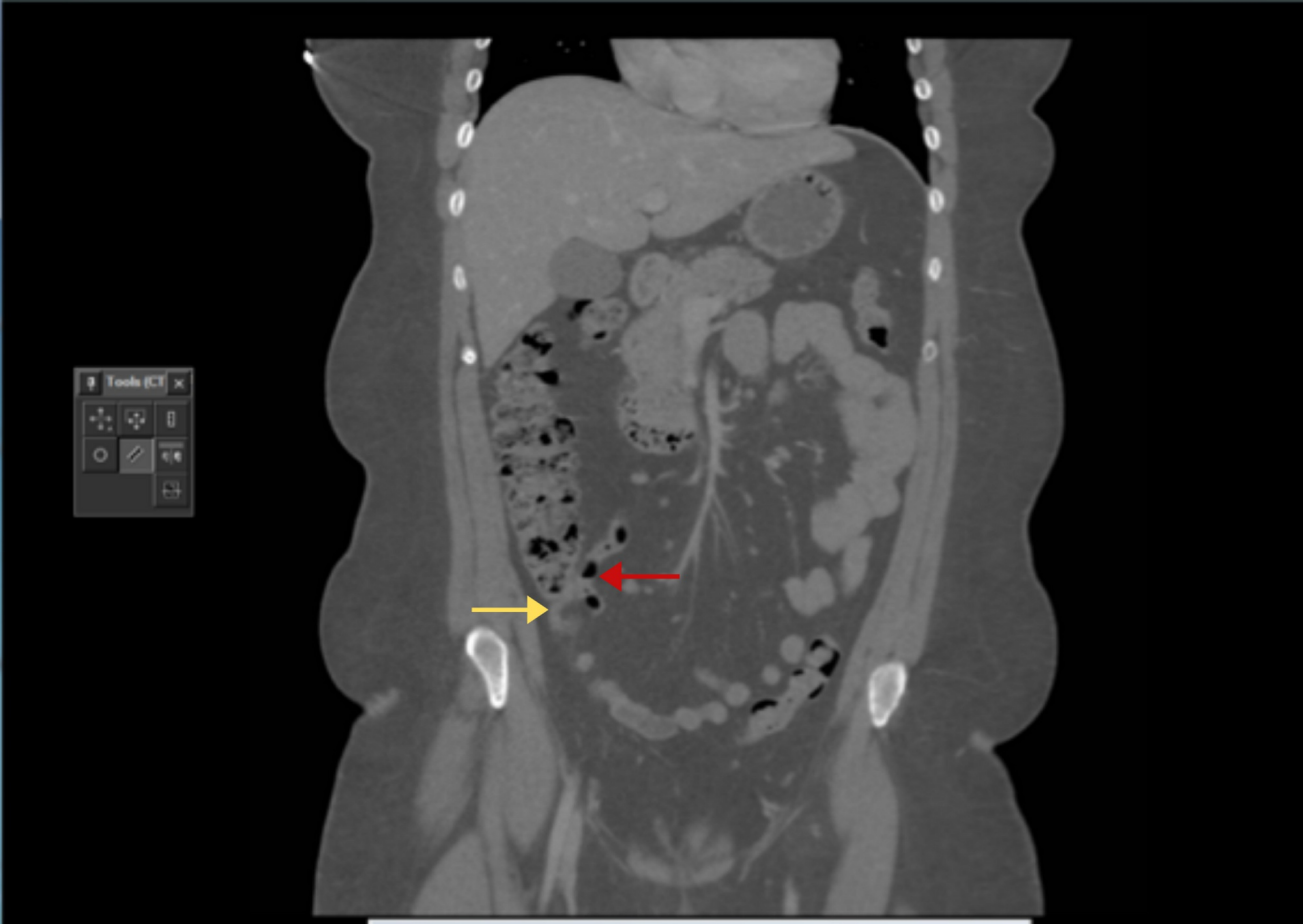
Coronal section of the CT scan showing a normal appendix (red arrow) with some fat stranding around the appendix (yellow arrow)

The first case is of a 43-year-old female with a body mass index (BMI) of 42 kg/m^2^, who presented to the emergency department with a three-day history of an acute onset right iliac fossa pain with no other associated symptoms. Her past medical history included hypertension and a previous laparoscopy for ovarian cyst. On admission, her vital signs were stable. Abdominal examination revealed an extremely tender right iliac fossa, with guarding and no rebound tenderness. Blood investigations showed normal inflammatory and biochemical markers. Abdominal CT revealed a normal appendix with some inflammatory stranding seen in the mesoappendix (Figure 1).

**Figure 2:**
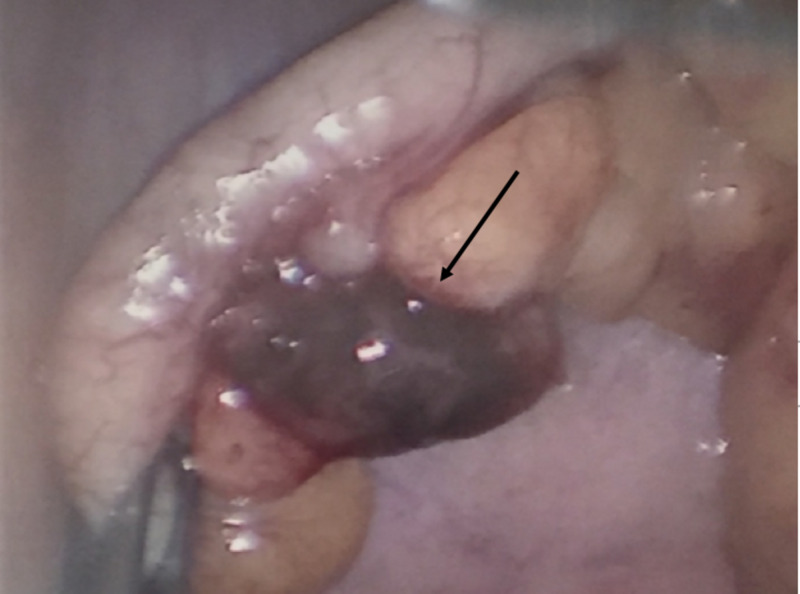
The intra-operative image showing a normal appendix with a gangrenous tortuous appendage next to a diverticulum of the appendix (arrow)

With the clinical diagnosis of an early acute appendicitis, an emergency diagnostic laparoscopy was performed. On laparoscopy, the appendix appeared normal, although a diverticulum of the appendix was noted with a tortuous and infarcted appendage in the mesoappendix (Figure 2).

**Figure 3:**
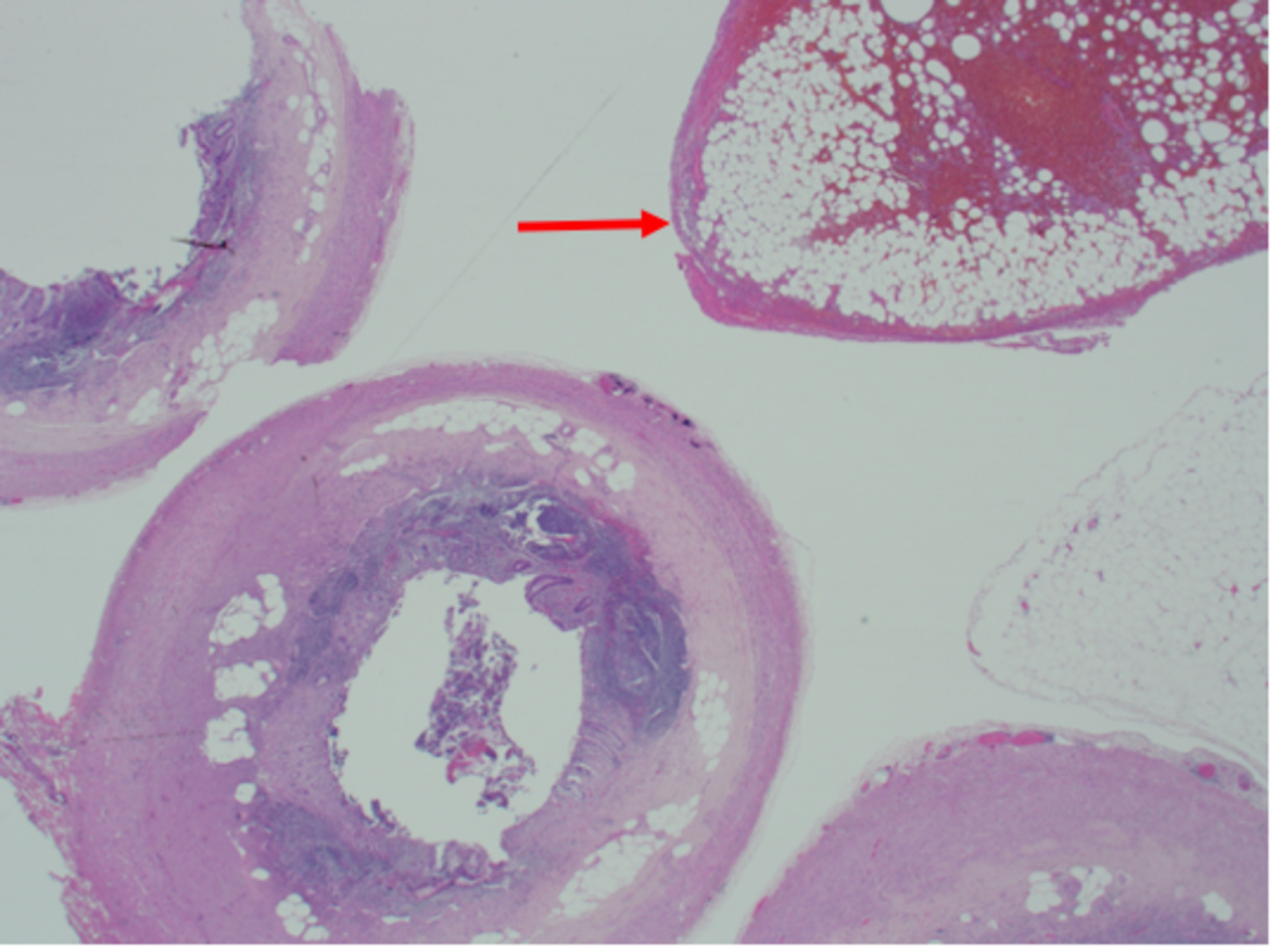
H&E section: transverse section of the appendix with a part of an epiploic appendage showing hemorrhagic infarction and fat necrosis (arrow) H&E, hematoxylin and eosin

The specimen was retrieved and sent for histopathology that showed a normal appendix with hemorrhagic infarction and fat necrosis of the EA (Figure 3).

**Figure 4:**
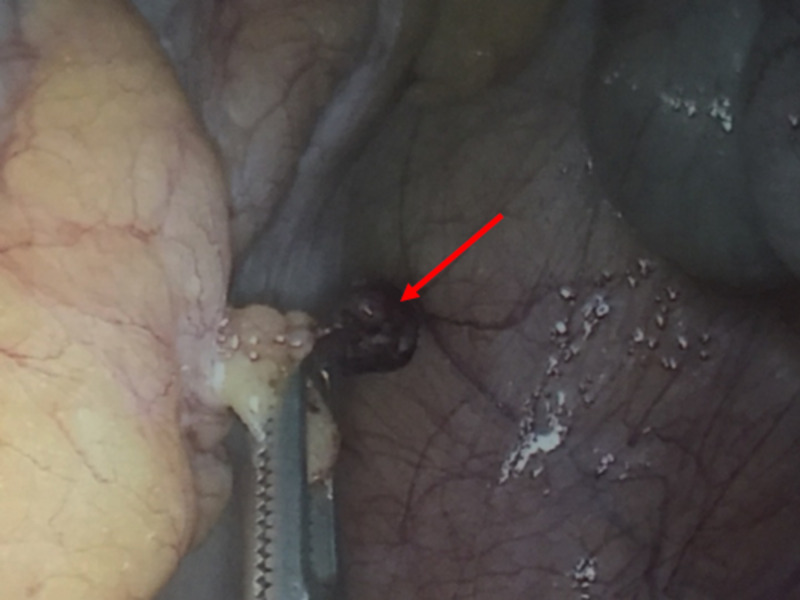
Laparoscopic intra-operative image showing a tortuous appendage in the parietal peritoneum in the right iliac fossa (arrow)

The second case is of a 22-year-old female with a BMI of 32 kg/m^2^ who was admitted with an acute onset right iliac fossa pain. Her past medical history was unremarkable. On examination, she had marked right iliac fossa tenderness and guarding with positive rebound tenderness. Rovsing and psoas signs were negative. With a clinical diagnosis of acute appendicitis, she underwent an emergency diagnostic laparoscopy that revealed a macroscopically normal-looking appendix. There was minimal serosanguinous fluid in the pelvis and no pelvic pathology was visualised on inspection. On further exploration, attached to the parietal peritoneum in the right iliac fossa, there was a pedunculated appendage that appeared infarcted and was therefore excised (Figure 4).

The patient was symptom free and comfortable on discharge. The histopathology report revealed a normal appendix.

## Discussion

Epiploic appendages are pendulous adipose outpouchings protruding from the serosal surfaces of the large bowel [2]. A normal adult human being can have about 50-100 epiploic appendages increasing progressively in size and number towards the recto-sigmoid [1].

These were anatomically first described in 1543 by Vesalius, but their clinical significance was not known until 1853 when a source of free intra-peritoneal loose bodies was questioned, and Virchow postulated that their detachment from the bowel surface might be the source [2,4]. Varying in length from 0.5 to 5 cm, epiploic appendages have one to two arterioles and a venule derived from vasa recti to form a vascular stalk. The exact function of epiploic appendages are questionable; they possibly act as a fat padding affording protection to the blood vessels. Some have attributed absorptive function and bacteriostatic function. It is also postulated that they may have a role in the immune response system.

EA can be either primary or secondary. The primary cause is either due to torsion or due to spontaneous venous thrombosis. Due to the pedunculated shape and tenuous blood supply, they are prone to spontaneous torsion. Torsion of epiploic appendages was first noted by Payr in 1902 [5]. It can result in ischemia and infarction of the appendage and can present as acute abdominal pain mimicking diverticulitis, appendicitis [6,7] or cholecystitis [8] depending on the location of the appendage. Primary EA presenting as a clinical entity due to de novo venous thrombosis of the draining venule was first described in 1956 by Lynn [9]. Inflammation of the adjacent organs like appendicitis, diverticulitis or cholecystitis can cause secondary EA [4].

The incidence of EA ranges from 2% to 7% in cases of provisional diagnosis of acute diverticulitis and 0.3% to 1% in suspected acute appendicitis cases [10]. Acute EA of the appendix, which is the inflammation of the epiploic appendages close to the vermiform appendix, is even rarer accounting for only 3% of all the EA cases [4], often masquerading as acute appendicitis. Most reports suggest male predominance [5,11,12] whereas some reports mentioned that it is more prevalent in female gender [13], but both our cases were females. An association with obesity, colonic diverticula, strenuous exercise and hernias has been noted in previous reports [12-14]. One of our patients had a BMI of 42. Adults are commonly afflicted, where the age of the reported cases ranges from 8 to 80 years with a peak incidence around 40 years [13,15].

Clinically, EA presents with acute colicky or constant abdominal pain mimicking diverticulitis, appendicitis or cholecystitis [2] depending on its site. Usually self-resolving, the pain lasts from a week to several months. Associated constitutional symptoms like fever are rare. Appetite is usually preserved and nausea and vomiting are uncommon. The clinical examination usually reveals localised rebound tenderness without rigidity. Furthermore, apart from pain from torsion or thrombosis, the inflammation of the appendage could later result in band adhesions presenting with intestinal obstruction. Some cases of primary band adhesion in virgin abdomens may represent a previously overlooked EA. When the pedicle atrophies as a result of aseptic fat necrosis, the appendage becomes an intra-peritoneal loose body, which may be detected at laparotomy/laparoscopy or on imaging. In rare instances, the loose body can re-attach to a peritoneal surface as in our second case, or rarely over the spleen as a parasitized appendix epiploica [16].

Given the non-specific laboratory investigations, imaging modalities are vital for diagnosis [3]. The hallmark ultrasonographic feature is the presence of a hyperechoic non-compressible pendulous tender mass adjacent to the bowel wall with absent flow on colour Doppler. The pathognomonic CT finding is a 2- to 4-cm oval-shaped fat density lesion with a central focal area of hyper-attenuation surrounded by inflammatory stranding [1,11]. Thickening of the adjacent parietal peritoneum may be seen. Unlike diverticulitis, the colonic wall usually does not show any signs of thickening [17]. A CT scan is more specific as compared to ultrasound for diagnosis. The natural history of EA is self-limiting, lasting up to two weeks with analgesic and anti-inflammatory medications. In any patient undergoing diagnostic laparoscopy, if there is a presence of serosanguinous fluid in the pelvis with no associated abdominal or pelvic pathology, the colon should be thoroughly examined for the presence of EA. When the surgeon encounters an EA on diagnostic laparoscopy in a symptomatic patient, the best treatment option is excision of the lesion [1,18,19].

## Conclusions

Although rare, EA should be an important differential diagnosis for the acute onset of localised abdominal pain without raised inflammatory markers. If suspected, an appropriate imaging modality, either abdominal ultrasound or CT scan, should be ordered to rule out EA. Early pre-operative diagnosis can prevent unnecessary hospitalisations, antibiotic therapy and surgical interventions. On diagnostic laparoscopy, in presence of serosanguinous free fluid in the abdomen and no associated pelvic pathology, a thorough examination of the colon should be done to look for any evidence of EA. Additionally, conservative management with non-steroidal anti-inflammatory drugs should be considered as its initial management in the absence of other complications.

## References

[1] Chu EA, Kaminer E (2018). Epiploic appendagitis: a rare cause of acute abdomen. Radiol Case Rep.

[2] Hwang JA, Kim SM, Song HJ (2013). Differential diagnosis of left-sided abdominal pain: primary epiploic appendagitis vs colonic diverticulitis. World J Gastroenterol.

[3] AL-Zawi ASA, Lazarevska A, Omer M, Lange-Ratajczak M, Abohamod E, Tan E (2018). Adenocarcinoma of appendix mimicking acute appendicitis: a case report and literature review. Eur J Pharm Med Res.

[4] Giannis D, Matenoglou E, Sidiropoulou MS, Papalampros A, Schmitz R, Felekouras E, Moris D (2019). Epiploic appendagitis: pathogenesis, clinical findings and imaging clues of a misdiagnosed mimicker. Ann Transl Med.

[5] Ross JA (1950). Vascular loops in the appendices epiploicae: their anatomy and surgical significance, with a review of the surgical pathology of appendices epiploicae. Br J Surg.

[6] Rashid A, Nazir S, Hakim SY, Chalkoo MA (2012). Epiploic appendagitis of caecum: a diagnostic dilemma. Ger Med Sci.

[7] Liveris A, Borenstein SH (2018). Cecal epiploic appendagitis mimicking appendicitis. J Pediatr Surg Case Rep.

[8] Lien WC, Lai TI, Lin GS, Wang HP, Chen WJ, Cheng TY (2004). Epiploic appendagitis mimicking acute cholecystitis. Am J Emerg Med.

[9] Dockerty MB, Lynn TE, Waugh JM (1956). A clinicopathologic study of the epiploic appendages. Surg Gynecol Obstet.

[10] Schnedl WJ, Krause R, Tafeit E, Tillich M, Lipp RW, Wallner-Liebmann SJ (2011). Insights into epiploic appendagitis. Nat Rev Gastroenterol Hepatol.

[11] Giambelluca D, Cannella R, Caruana G (2019). CT imaging findings of epiploic appendagitis: an unusual cause of abdominal pain. Insights Imaging.

[12] Son HJ, Lee SJ, Lee JH (2002). Clinical diagnosis of primary epiploic appendagitis: differentiation from acute diverticulitis. J Clin Gastroenterol.

[13] Almeida AT, Melão L, Viamonte B, Cunha R, Pereira JM (2009). Epiploic appendagitis: an entity frequently unknown to clinicians - diagnostic imaging, pitfalls, and look-alikes. AJR Am J Roentgenol.

[14] Singh AK, Gervais DA, Hahn PF, Sagar P, Mueller PR, Novelline RA (2005). Acute epiploic appendagitis and its mimics. Radiographics.

[15] Fraser JD, Aguayo P, Leys CM, St. Peter SD, Ostlie DJ (2009). Infarction of an epiploic appendage in a pediatric patient. J Pediatr Surg.

[16] Sand M, Gelos M, Bechara FG, Sand D, Wiese TH, Steinstraesser L, Mann B (2007). Epiploic appendagitis - clinical characteristics of an uncommon surgical diagnosis. BMC Surg.

[17] van Breda Vriesman AC, Puylaert JB (2002). Epiploic appendagitis and omental infarction: pitfalls and look-alikes. Abdom Imaging.

[18] Chowbey PK, Singh G, Sharma A, Khullar R, Soni V, Baijal M (2003). Torsion of appendices epiploicae presenting as acute abdomen: laparoscopic diagnosis and therapy. Indian J Gastroenterol.

[19] Golash V, Willson PD (2005). Early laparoscopy as a routine procedure in the management of acute abdominal pain: a review of 1,320 patients. Surg Endosc.

